# Health Care Professionals’ Interest in Vaccination Training in Switzerland: A Quantitative Survey

**DOI:** 10.3389/ijph.2022.1604495

**Published:** 2022-11-30

**Authors:** Pia Lucas Ramanathan, Nadja Baldesberger, Léna G. Dietrich, Camilla Speranza, Alyssa Lüthy, Andrea Buhl, Martina Gisin, Roswitha Koch, Dunja Nicca, L. Suzanne Suggs, Benedikt M. Huber, Michael J. Deml, Philip E. Tarr

**Affiliations:** ^1^ University Department of Medicine and Infectious Diseases Service, Kantonsspital Baselland, Bruderholz, Switzerland; ^2^ University of Basel, Basel, Switzerland; ^3^ Pharmaceutical Care Research Group, Basel, Switzerland; ^4^ Institute of Public Health, and Institute of Communication and Public Policy, Università della Svizzera italiana, Lugano, Switzerland; ^5^ Swiss Tropical and Public Health Institute (Swiss TPH), Basel, Switzerland; ^6^ Department of Obstetrics and Gynecology, University Hospital of Basel, Basel, Switzerland; ^7^ Swiss Nurses Association, Bern, Switzerland; ^8^ Epidemiology, Biostatistics and Prevention Institute, University of Zurich, Zurich, Switzerland; ^9^ Swiss School of Public Health, Zurich, Switzerland; ^10^ Center for Integrative Pediatrics, Department of Pediatrics, Fribourg Hospital HFR, Fribourg, Switzerland; ^11^ Faculty of Science and Medicine, University of Fribourg, Fribourg, Switzerland; ^12^ Institute of Sociological Research, Department of Sociology, University of Geneva, Geneva, Switzerland

**Keywords:** vaccine hesitancy, communication, healthcare professional, vaccine knowledge, medical training

## Abstract

**Objectives:** Health care professionals (HCPs) play an important role for patients’ vaccination decisions. To counsel patients/clients appropriately, HCPs need current factual knowledge about vaccines and strong communication skills.

**Methods:** We conducted an online survey with physicians, pharmacists, nurses, and midwives in Switzerland (01.11.2020–31.03.2021). We evaluated: 1) interest in vaccination knowledge and counseling training; 2) vaccination recommendation practices; 3) experience with vaccination counseling/administration; 4) comfort level in addressing vaccine hesitancy (VH); 5) perspectives on patient/client VH, delays, and refusals.

**Results:** In total, 1,933 practicing HCPs responded (496 physicians, 226 pharmacists, 607 nurses, 604 midwives). 43% physicians, 31% pharmacists, 15% nurses, and 23% midwives felt comfortable counseling VH patients/clients. 96% physicians, 98% pharmacists, 85% nurses, and 91% midwives were interested in additional vaccination-related training. All professionals mentioned safety, efficacy, and side effects as topics of most interest for additional training.

**Conclusion:** Results demonstrate a high interest among HCPs for additional vaccination-related training. In addition to factual information about vaccination, such training will likely benefit from a communication component, given the low rates of comfort reported by HCPs when counseling VH patients/clients.

## Introduction

To adequately respond to vaccination questions of patients and their caregivers, it is important that health care professionals (HCPs) have sufficient vaccination knowledge and communication skills. HCPs play a key role in influencing vaccination decisions, and research consistently shows that patients/clients cite them as the most trusted source in vaccine decisions (1–4). Despite HCPs generally having confidence in vaccines, several studies show that some HCPs feel uneasy when addressing vaccination questions in clinical practice and are not always able to answer detailed questions about vaccine safety, ingredients, or necessity (5–10). In addition, the type of vaccination under discussion (e.g., influenza) can influence HCPs’ level of confidence discussing or recommending the vaccine (11, 12). HCPs in high-income countries have expressed interest in obtaining additional training about vaccines and vaccine communication in order to improve their vaccination counseling (13, 14). Karafillakis and Larson (2018) argued that HCP training interventions that incorporate HCP’s perspectives would likely result in HCPs having more confidence in their own vaccination knowledge and communication skills (15).

A randomized trial conducted in 2020 with US physicians-in-training found that obtaining in-depth training on vaccination as well as advanced communication training can improve pediatric and family medicine residents’ confidence in addressing parental vaccination concerns (13). Research has also shown that physicians sometimes consider vaccine hesitant (VH) parents to be difficult patients/clients and vaccination consultations can pose challenges and elicit dilemmas about their professional roles and responsibilities (14, 16–18). Primary care physicians reported that parental vaccine safety concerns are becoming more frequent (16).

In Switzerland, there is a widespread use of complementary medicine (CM) (19, 20) and many HCP have additional trainings in CM (21, 22). For example, practicing physicians are trained in biomedicine (i.e. conventional, mainstream medicine) and some obtain CM training too. Knowing that CM plays a role in the context of vaccination and VH (23), there might also be different attitudes and needs of HCPs whether or not they have additional training in CM. Research shows that CM providers play an important role in vaccination counseling in Switzerland (14, 24). The use of CM and is common among 25%–50% of the population. Contrary to common preconceptions, CM providers are not categorically opposed to vaccination (25). Rather, research has shown how they prefer individualized, patient-centered vaccination counseling that addresses the pros and cons of vaccination, emphasizes the autonomy of parents in their medical decisions, and involves them in vaccination decisions (26, 27).

Given these considerations, we aimed to understand the needs of HCPs for additional vaccination knowledge and communication training in Switzerland in the setting of the Swiss National Research Program 74 (NRP74) on VH (24). With this aim, we developed and administered an online survey to assess interest in training regarding vaccination knowledge and communication needs among HCPs across Switzerland. Survey results will inform the development of interventions to address VH in clinical practice through HCP education efforts. Survey results related to vaccine mandates have previously been published (28).

### The Swiss Healthcare System

In Switzerland, the Federal Office of Public Health develops and publishes a national vaccination plan together with the Federal Commission for Immunization. HCPs are meant to contribute to the implementation of the vaccination plan by informing parents/patients about vaccination recommendations. As vaccinations are voluntary, parents/patients are not required to adhere to official recommendations.

In Switzerland, vaccination rates are relatively high overall (27), but they vary widely, with measles and human papilloma viruses (HPV) vaccination rates being higher in French-and Italian-speaking regions than in German-speaking regions of Switzerland. Moreover, HPV vaccination rates are higher where the vaccine is offered through schools (24, 29, 30).

## Methods

### Survey Development

We developed an online survey to assess interest in training regarding vaccination knowledge and communication needs among HCPs across Switzerland. In a first step, we assembled potentially relevant questions and divided them into appropriate categories (16–18). In discussions among our multidisciplinary team (including biomedicine, CM, sociology, anthropology, epidemiology, public health, social marketing, and communication sciences) we condensed the survey, with the goal of making it concise and appropriate for the different health professions. Qualtrics software (Qualtrics XM, Provo, Utah, US) was used as the online platform. The survey was translated from English into three Swiss national languages (German, French, and Italian) by bilingual research team members. The surveys were then administered in these four languages, with respondents choosing their preference at the beginning of the survey. We piloted the survey in all national languages with four participants from each target group (physicians, pharmacists, nurses, and midwives), and then discussed and adjusted the survey wording accordingly in each language. We display study results in English.

### Survey Content

The survey contained questions addressed to each HCP group, with common items and branching logic for specific items tailored to the type of provider: 29 (physicians), 28 (pharmacists, nurses), and 25 (midwives), respectively. Hospital pharmacists were excluded from the survey due to their infrequent contact with clients around the topic of vaccination. Medical practice assistants and medical students were excluded from the survey because they only administer vaccinations at the direction of the supervising physician. The survey included questions about the following topics (1): interest in obtaining additional training related to vaccination knowledge and counseling, particularly in relation to the Swiss vaccination schedule (2); vaccination counseling frequency and practices (3); experience with vaccination counseling and administration (4); level of comfort when dealing with VH patients/clients (5); perspectives on VH, vaccine delays and refusals. We included demographic and background questions (age, gender, profession, location of work, type of work, position, additional designation, and year of graduation), questions regarding interest in preferred training modalities (in-person workshops, online interactive workshops, online lectures, written material), and desired training content (vaccine safety, efficacy, side effects, vaccine-preventable diseases, vaccine components, immunology, communication with VH individuals, shared decision making and details of the Swiss vaccination schedule) (Supplementary File). Not all survey questions were asked to each HCP group, due to the fact that some HCP groups are rarely exposed to certain topics, e.g., the question about their recommendation for HPV vaccine was not asked to midwives.

To assess participant vaccination recommendation behavior a 5-point Likert scale (1: “do not encourage vaccination at all” to 5: “encourage vaccination completely”) was used. We asked participants if they encouraged patients/clients to follow the official recommendations regarding childhood vaccinations and HPV vaccine for adolescents. We also asked about personal experiences with vaccination counseling and administration. HCPs chose from a list of statements and indicated how often they counsel VH patients/clients and how often they answer questions about vaccination in their friend/acquaintance networks.

### Survey Administration

The survey was available online from 1 November 2020 to 31 March 2021 and most participants completed it in 5–8 min. We invited physicians, pharmacists in private pharmacies, nurses, and midwives through mailing lists of key professional societies. Membership in these societies is not compulsory in Switzerland, but our team’s professional experience suggests membership is nearly universal. The professional societies invited their members to participate through email (nurses, midwives), through their monthly newsletter (pharmacists) or society journals (physicians). Participating societies included Kinderärzte Schweiz (31), Swiss Society of Pediatrics (pédiatrie suisse) (32), Swiss Society of General and Internal Medicine (33), the Swiss Pharmacists Association (pharmaSuisse) (34), Swiss Association of Nurses (35), and Swiss Association of Midwives (36). We asked professional societies to send out one email reminder to their members 3–4 weeks after the first invitation. Participation in the survey was voluntary and anonymous. The study was approved by the local ethics committee (Ethikkommission Nordwest- und Zentralschweiz), and participants provided informed consent (project-ID: 2017-00725).

### Data Analysis

To calculate approximate response rates among the four professional groups, we divided the number of participants by the number of members of each professional society indicated on their respective websites (31–36). We tabulated and analyzed the data using IBM SPSS for Mac statistical package version 27. We report the observations as percentages and used chi-square test to test for associations between each pair of categorical variables. The expected frequencies of each category of each variable have resulted to be greater than five. For this reason, the results have statistical power, i.e., it is likely that the test may detect a genuine effect. We tested for significant differences across the four professions (physicians, pharmacists, nurses, and midwives).

Specifically, we checked for associations between level of comfort in counseling VH patients/clients and

(1) whether or not the HCP has additional training in CM.
(2) years of professional experience of the HCPs.
(3) the frequency of HCP encounters with these individuals;

We also checked for associations between whether the HCP has additional training in CM and

(1) HCPs reporting patients/clients stop coming to them due to a disagreement about vaccination
(2) HCPs reporting patients/clients deciding to come to them due to a disagreement about vaccination with another HCP.

We calculated the years of professional experience by subtracting the year of graduation from the year of the survey. We conducted one-way analysis of variance (ANOVA) to determine if there were significant differences between the means of the three different groups for level of comfort when dealing with VH patients/clients (comfortable, neither comfortable nor uncomfortable, uncomfortable). Subsequently, we ran a post hoc test procedure (Hochberg’s GT2, checking with the Games–Howell procedure) to find out which groups differ.

## Results

### Respondents

1,933 participants responded to the survey, including 496 physicians, 226 pharmacists, 607 nurses, and 604 midwives. The response rate was 4.4% (1,933 respondents among 44,290 society members). The response rate was 496/9,390 (5.3%) among physicians, 226/6,700 (3.4%) among pharmacists, 607/25,000 (2.4%) among nurses, and 604/3,200 (19%) among midwives.

Participant characteristics are shown in Table 1. Respondents were located in all 3 main language regions of Switzerland: German (*n* = 1,627, 86%), French (*n* = 232, 12%), and Italian (*n* = 42, 2%). The majority of participants were in the 41–60-year age category. There were more female than male participants, particularly among nurses and midwives. Among physicians, 75% worked in pediatrics and most had obtained a specialist degree. The majority of nurses worked in adult medicine, with ∼66% working in the hospital and with direct patient contact. The majority of midwives were self-employed freelancers. Between 10% (physicians) and 45% (midwives) of participants had additional training in CM.

**TABLE 1:** Sample characteristics (Switzerland. 2020–2021).

	Physicians (N = 496)	Pharmacists (N = 226)	Nurses (N = 607)	Midwives (N = 604)	Swiss population (N = 8,736,500)
Age					
≤40 years	125 (25%)	92 (41%)	229 (38%)	266 (44%)	4,018,790 (46%)
41–60 years	292 (59%)	114 (50%)	340 (56%)	292 (48%)	3,057,775 (35%)
≥61 years	79 (16%)	20 (9%)	38 (6%)	46 (8%)	1,659,935 (19%)
Gender					
Female	332 (67%)	189 (84%)	552 (92%)	604 (100%)	4,403,196 (50%)
Male	164 (33%)	37 (16%)	51 (8%)	0 (0%)	4,333,304 (50%)
Language region in Switzerland					
German	380 (78%)	188 (84%)	530 (89%)	529 (89%)	5,503,995 (63%)
French	93 (19%)	25 (11%)	62 (10%)	52 (9%)	2,009,395 (23%)
Italian	14 (3%)	10 (4%)	5 (1%)	13 (2%)	698,920 (8%)
Field of work					
Pediatrics	387 (75%)	N.A.^a^	145 (21%)	N.A.	
General medicine (adults):	57 (11%)	N.A.^a^		N.A.	
Adults (surgery, medicine):		N.A.^a^	272 (40%)	N.A.	
Other (e.g. gynecology, psychiatry):	75 (14%)	N.A.^a^	258 (39%)	N.A.	
Place of work	Private Practice: 422 (79%)	N.A.^a^	Hospital: 396 (66%)	Freelance:	
	Hospital:		Outpatient/visiting nurse/freelance:	420 (42%)	
	114 (21%)		146 (24%)	Labor ward:	
			Other (e.g. rehabilitation clinic): 55 (10%)	221 (22%)	
				Other (e.g. postnatal ward, prenatal ward): 351 (35%):	
Position					
Finished specialist training	445 (93%)				
In training	36 (7%)			17 (2%)	
Employer		142 (64%)		257 (35%)	
Management function		81 (36%)	148 (20%)	44 (6%)	
Work with patient contact			506 (69%)		
Education			58 (8%)		
Research			20 (3%)		
Self-employed				422 (57%)	
Accreditation or additional training in complementary medicine	49 (10%)	47 (22%)	107 (18%)	269 (45%)	
Type of complementary medicine^b^					
Anthroposophic medicine	12 (24%)	8 (14%)	10 (10%)	18 (4%)	
Acupuncture	0 (0%)	0 (0%)	4 (4%)	136 (33%)	
Homeopathy	10 (20%)	29 (49%)	10 (10%)	104 (25%)	
Phytotherapy	8 (16%)	11 (19%)	9 (9%)	43 (10%)	
Traditional Chinese Medicine	8 (16%)	0 (0%)	2 (2%)	35 (8%)	
Other (e.g., Craniosacral therapy, spagyric, massage, Bach flowers, aromatherapy)	13 (25%)	11 (19%)	69 (66%)	78 (19%)	

^a^ not applicable because only pharmacists in private practice were invited to participate.

^b^ multiple answers possible.

*N.A., not applicable.

### Interest in Additional Vaccination Knowledge and Counseling Training and Preferred Training Modalities

**FIGURE 1:**
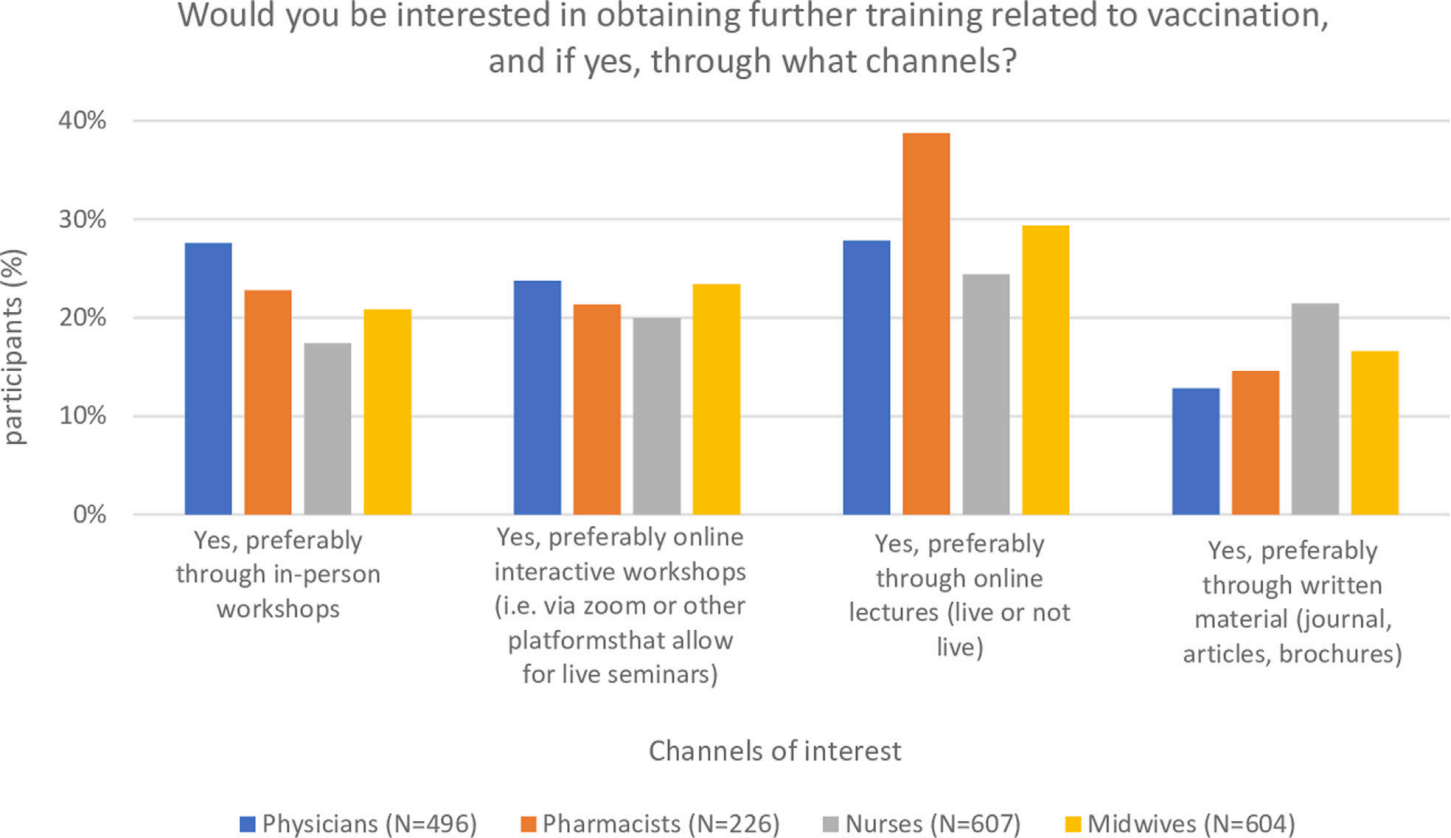
Preferred training modalities of further training related to vaccination (Switzerland. 2020–2021).

**FIGURE 2:**
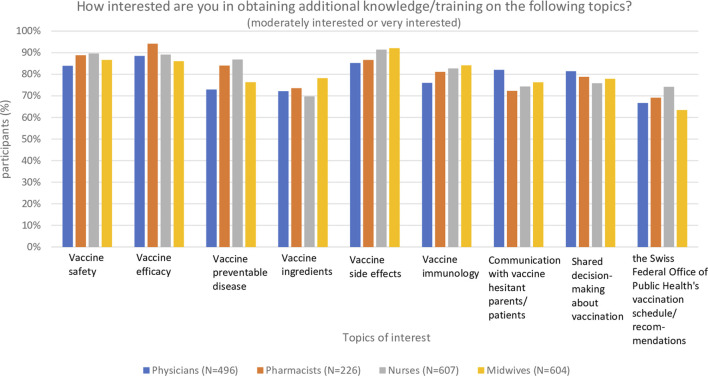
Preferred training contents of further training related to vaccination (Switzerland. 2020–2021).

Most participants were interested in obtaining additional training related to vaccination: 96% of physicians, 98% of pharmacists, 85% of nurses and 91% of midwives. Regarding the preferred training modality for additional vaccination training (Figure 1), HCPs mentioned in-person workshops, online interactive workshops, and online lectures with similar frequency, except for pharmacists who indicated a slight preference for online lectures. All vaccination topics offered in the survey were of interest to a large majority of HCPs (Figure 2), with limited differences between topics and between HCP groups. The topics for which >80% of respondents from each HCP group expressed additional training interest were vaccine safety, efficacy, and side effects.

### Vaccination Recommendation and Administration

0% of physicians, 3% of pharmacists, 17% of nurses and 6% of midwives were unfamiliar with the Swiss vaccination plan for children. 88% of physicians, 68% of pharmacists, 37% of nurses, and 14% of midwives encouraged patients/clients to follow the official childhood vaccination recommendations (Table 2). For HPV-vaccine, 74% of physicians, 44% of pharmacists, and 23% of nurses encouraged patients/clients to follow vaccination recommendations (Table 2). 3% of physicians, 16% of pharmacists and 37% of nurses were unfamiliar with the HPV vaccination schedule. 79% of physicians and 56% of pharmacists were somewhat or completely satisfied with how the health authorities communicate about the official vaccination schedule. Only 34% of nurses and 27% of midwives shared this view (Table 2).

**TABLE 2:** Vaccination recommendation and administration (Switzerland. 2020–2021).

	Totals	Responses for different HCP professions				*p*-value^a^
		Physicians	Pharmacists	Nurses	Midwives	
To what extent do you encourage parents/patients to follow the official Swiss vaccination schedule for children?	*N* = 1919	*N* = 494	*N* = 223	*N* = 602	*N* = 600	
1 = not at all	127 (7%)	2 (0%)	11 (5%)	60 (10%)	54 (9%)	χ2 (8) = 830.06 *p = <*0.001
2 = slightly	99 (5%)	6 (1%)	6 (3%)	25 (4%)	62 (10%)	
3 = moderately	301 (16%)	10 (2%)	13 (6%)	73 (12%)	205 (34%)	
4 = mostly	360 (19%)	41 (8%)	36 (16%)	122 (20%)	161 (27%)	
5 = completely	890 (46%)	435 (88%)	151 (68%)	221 (37%)	83 (14%)	
I am not familiar with the Swiss vaccination plan	142 (7%)	0 (0%)	6 (3%)	101 (17%)	35 (6%)	
To what extent do you encourage parents/patients to follow the official Swiss human papillomavirus (HPV) vaccination schedule for adolescents and young adults?	*N* = 1314	*N* = 49*5*	*N* = 224	*N* = 595	N.A.	
1 = not at all	98 (7%)	6 (1%)	20 (9%)	72 (12%)	N.A.	χ2 (8) = 2436.83 *p = <*0.000
2 = slightly	41 (3%)	11 (2%)	6 (3%)	24 (4%)	N.A.	
3 = moderately	103 (8%)	21 (4%)	15 (7%)	67 (11%)	N.A.	
4 = mostly	199 (15%)	78 (16%)	49 (22%)	72 (12%)	N.A.	
5 = completely	602 (46%)	366 (74%)	99 (44%)	137 (23%)	N.A.	
I am not familiar with the Swiss vaccination plan	271 (21%)	13 (3%)	35 (16%)	223 (37%)	N.A.	
How satisfied are you with how the Swiss Federal Office of Public Health communicates the official Swiss vaccination schedule?	*N* = 1919	*N* = 494	*N* = 223	*N* = 602	*N* = 600	
Completely dissatisfied	72 (4%)	8 (2%)	2 (1%)	23 (4%)	39 (7%)	χ2 (8) = 451.2 *p = <*0.001
Somewhat dissatisfied	263 (14%)	24 (5%)	27 (12%)	88 (15%)	124 (21%)	
Neither satisfied nor dissatisfied	571 (30%)	72 (15%)	64 (29%)	193 (32%)	242 (40%)	
Somewhat satisfied	619 (32%)	230 (47%)	94 (42%)	164 (27%)	131 (22%)	
Completely satisfied	262 (14%)	156 (32%)	31 (14%)	45 (7%)	30 (5%)	
Do you administer vaccination?	*N* = 1900	*N* = 492	*N* = 218	*N* = 603	*N* = 587	
Yes	1113 (59%)	478 (97%)	165 (76%)	348 (58%)	138 (23%)	χ2 (8) = 647.66 *p = <*0.001
No	787 (41%)	15 (3%)	53 (24%)	255 (42%)	464 (77%)	
How often do you vaccinate?	*N* = 1117	*N* = 468	*N* = 171	*N =* 342	*N* = 136	
At least 1x/day	326 (29%)	310 (66%)	2 (1%)	12 (4%)	2 (1%)	χ2 (8) = 1814.31 *p = <*0.000
At least 1x/week	202 (18%)	97 (21%)	77 (45%)	22 (6%)	6 (4%)	
At least 1x/month	179 (16%)	32 (7%)	78 (46%)	51 (15%)	18 (13%)	
Less often than 1x/month	410 (37%)	29 (6%)	14 (8%)	257 (75%)	110 (81%)	
How often do you answer questions from patients/clients about vaccines?	*N =* 1887	*N* = 482	*N* = 223	*N* = 584	*N* = 598	
At least 1x/day	334 (18%)	282 (59%)	11 (5%)	31 (5%)	10 (2%)	χ2 (8) = 1037.26 *p = <*0.001
At least 1x/week	452 (24%)	127 (26%)	81 (36%)	76 (13%)	168 (28%)	
At least 1x/month	474 (25%)	52 (11%)	81 (36%)	116 (20%)	225 (38%)	
Less often than 1x/month	627 (33%)	21 (4%)	50 (22%)	361 (62%)	195 (33%)	
How often do you answer questions about vaccinations from friends/acquaintances (i.e. outside of work)?	*N* = 1902	*N* = 487	*N* = 223	*N =* 593	*N* = 599	
At least 1x/day	40 (2%)	14 (3%)	2 (1%)	22 (4%)	2 (0%)	χ2 (8) = 217.16 *p = <*0.001
At least 1x/week	289 (15%)	67 (14%)	45 (20%)	145 (24%)	32 (5%)	
At least 1x/month	648 (34%)	215 (44%)	109 (49%)	166 (28%)	158 (26%)	
Less often than 1x/month	925 (49%)	191 (39%)	67 (30%)	260 (44%)	407 (68%)	
Have you ever refused care to parents/patients because he/she asked for vaccination later than recommended or refused vaccination altogether?	N = 485	N = 485	N.A.	N.A.	N.A.	
Yes	50 (10%)	50 (10%)	N.A.	N.A.	N.A.	N.A.
No	415 (86%)	415 (86%)	N.A.	N.A.	N.A.	
I am not sure	20 (4%)	20 (4%)	N.A.	N.A.	N.A.	
Have any patients/clients stopped coming to you for consultations due to a disagreement with you about vaccination?	*N* = 708	*N =* 486	*N =* 222	N.A.	N.A.	
Yes	142 (23%)	141 (29%)	1 (0%)	N.A.	N.A.	χ2 (8) = 2094.89 *p = <*0.000
No	413 (58%)	240 (49%)	173 (78%)	N.A.	N.A.	
I am not sure	153 (22%)	105 (22%)	48 (22%)	N.A.	N.A.	
Have any patients/clients decided to come to you for consultations due to a disagreement about vaccination with another healthcare provider?	*N* = 708	*N* = 487	*N* = 221	N.A.	N.A.	
Yes	221 (31%)	200 (41%)	21 (10%)	N.A.	N.A.	χ2 (8) = 2070.1 *p = <*0.000
No	291 (41%)	164 (34%)	127 (57%)	N.A.	N.A.	
I am not sure	196 (28%)	123 (25%)	73 (33%)	N.A.	N.A.	
When parents/patients disagree with my recommendations about vaccination, I feel it shows a lack of respect for my medical expertise/nursing competence/professional competence	*N* = 1311	*N* = 422	*N* = 183	*N* = 362	*N* = 344	
I agree	36 (3%)	19 (5%)	7 (4%)	10 (3%)	0 (0%)	χ2 (8) = 162.01 *p = <*0.001
Neither agree, nor disagree	272 (20%)	91 (22%)	49 (27%)	78 (22%)	54 (16%)	
I disagree	1003 (77%)	312 (74%)	127 (69%)	274 (76%)	290 (84%)	

^a^ Chi square test of independence: Statistically significant at *p* < .05 significance level.

Table 2 provides details about vaccine counseling and administration. There was a significant association between professional group and each of the questions asked in Table 2. 59% of physicians reported that they answer questions about vaccines from patients/clients on a daily basis, whereas other HCP groups reported doing so less frequently. 28% of nurses answered questions about vaccines from friends/acquaintances at least once a week, whereas the other HCPs did so less frequently.

The majority of physicians (97%), pharmacists (76%), and nurses (58%) reported that they administer vaccines, but only 23% of midwives did so (Table 2). Physicians and pharmacists were more likely to encourage patients to follow the official childhood and HPV vaccination schedule than nurses (and midwives regarding childhood vaccines).

10% of physicians reported having previously refused care to parents/patients who refused vaccines or who asked for a vaccine to be administrated later than officially recommended (Table 2)*.* 29% of physicians and 1 pharmacist (0.5%) reported that patients/clients sometimes stopped coming to them because of a disagreement around vaccination recommendations (Table 2). In contrast, 41% of physicians and 10% of pharmacists reported that patients/clients sometimes came to see them specifically due to a disagreement around vaccination with another HCP (Table 2).

16% of the HCP with additional CM training indicated that patients sometimes stopped coming to them due to a disagreement about vaccination with them, compared to the 27% of the non-CM trained HCPs (*p* = 0.038). Conversely, 50% of CM-trained providers indicated that patients sometimes came to them due to a disagreement about vaccination with another HCP, compared to the 42% of the non-CM providers (*p* = 0.038). Less than 5% of HCPs across all professions agreed with the statement that it shows a lack of respect when patients/clients disagree with their recommendations (Table 2).

### Level of Comfort in Addressing VH Patients/Clients

42% of physicians encountered VH patients/clients at least once a week. In contrast, more than 85% of pharmacists, nurses and midwives encountered VH patients/clients once a month or less often. 43% of physicians, 31% of pharmacists, 15% of nurses and 23% of midwives felt comfortable counseling VH patients/clients (Table 3). 2% or less of all HCP groups reported they preferred to no longer see VH patients/clients.

**TABLE 3:** Encounter frequency, level of comfort when counseling vaccine hesitant patients/clients (Switzerland. 2020–2021).

	Totals	Responses			
		Physicians	Pharmacists	Nurses	Midwives
How often do you encounter vaccine hesitant patients/clients?	*N =* 1890	*N* = 487	*N* = 223	*N* = 584	*N* = 596
At least 1x/day	57 (3%)	39 (8%)	1 (0%)	15 (3%)	2 (0%)
At least 1x/week	335 (18%)	168 (34%)	31 (14%)	62 (11%)	74 (12%)
At least 1x/month	556 (29%)	183 (38%)	65 (29%)	109 (19%)	199 (33%)
Less often than 1x/month	942 (50%)	97 (20%)	126 (57%)	398 (68%)	321 (54%)
How comfortable are you counseling vaccine hesitant patients/clients?	*N* = 1871	*N* = 484	*N* = 221	*N* = 570	*N* = 596
Comfortable	496 (27%)	206 (43%)	68 (31%)	83 (15%)	139 (23%)
Neither comfortable nor uncomfortable	898 (48%)	187 (39%)	117 (53%)	264 (46%)	330 (55%)
Uncomfortable	292 (16%)	81 (17%)	23 (10%)	100 (18%)	88 (15%)
It does not matter, as I prefer to no longer see these patients/clients	23 (1%)	8 (2%)	3 (1%)	7 (1%)	5 (1%)
Not applicable	162 (9%)	2 (0%)	10 (5%)	116 (20%)	34 (6%)

HCPs with CM training felt more comfortable in counseling VH patients/clients, compared to HCPs without CM training (Table 4). Along the same lines, more non-CM providers felt uncomfortable compared to the CM providers. With increasing age, HCPs reported feeling more comfortable counseling VH patients/clients, and along these lines, with increasing age fewer HCPs reported feeling uncomfortable (Table 4). In addition to age, HCPs feeling comfortable counseling VH parents/patients had more mean years of professional experience (23.9 +/− 11.3 years) compared to HCPs who felt neither comfortable nor uncomfortable (20.6 +/− 11.6 years) or uncomfortable (15.5 years +/− 11.3 years). There was a significant effect of years of experience on levels of comfort in counseling VH patients/clients, F (2, 1662) = 49.046.12, *p* < 0.001. The post hoc test procedure showed that there is a significant effect for each category. Additionally, there was an association between the frequency with which HCPs encounter VH patients/clients, and their indication of feeling comfortable in counseling them (Table 4). And with decreasing encounter frequency, HCP indicated feeling less comfortable in counseling VH patients/clients.

**TABLE 4:** Association between level of comfort in counseling vaccine hesitant patients/clients and whether or not the health care professional has additional training in complementary medicine, ages of the health care professionals, and the frequency of health care professional encounters these individuals (Switzerland. 2020–2021).

Comfort:	Comfortable counseling vaccine hesitant parents/patients	Neither comfortable nor uncomfortable counseling vaccine hesitant parents/patients	Uncomfortable counseling vaccine hesitant parents/patients	*p*-value^a^
Variable				
Additional training in CM	145 (34.5%)	221 (52.6%)	54 (12.9%)	χ2 (2) = 11.588 *p =* 0.003
No additional training in CM	348 (27.7%)	673 (53.5%)	237 (18.8%)	
Years of experience				
≤30 years	28 (15%)	90 (48.1%)	69 (36.9%)	χ2 (8) = 115.394 *p = <*0.001^a^
31–40 years	83 (20%)	243 (58.4%)	90 (21.6%)	
41–50 years	143 (32%)	235 (52.6%)	69 (15.4%)	
51–60 years	168 (35.7%)	251 (53.4%)	51 (10.9%)	
≥60 years	72 (43.9%)	79 (48.2%)	13 (7.9%)	
Frequency of encounters with vaccine hesitant patients				
At least once a day	30 (55.6%)	18 (33.3%)	6 (11.1%)	χ2 (6) = 112.575 *p = <*0.001^a^
At least once a week	140 (43.8%)	144 (45%)	36 (11.3%)	
At least once a month	186 (34.6%)	275 (51.1%)	77 (14.3%)	
Less often than once a month	137 (17.8%)	460 (59.7%)	173 (22.5%)	

^a^ Chi square test of independence: Statistically significant at *p* < 0.05 significance level.

## Discussion

This study of HCPs’ interest in additional training on vaccination knowledge and communication with VH patients/clients has three main findings. First, a large majority of all HCP groups are interested in additional training related to vaccination knowledge and communication. Despite a large proportion of nurse (42%) and midwife (77%) respondents not administering vaccinations, these groups nonetheless expressed high interest in additional vaccination training. As potential trusted sources of vaccination information, their interest in training should be taken into consideration. All topics proposed were of high interest, with vaccine safety, efficacy, and side effects being the topics mentioned most often by all four HCP groups. Such a high interest in obtaining additional training was also observed in a 2020 US study in which 88% of physicians-in-training said they would like to learn more about recommended childhood vaccines (37). Regarding the channels through which HCP preferred to obtain additional vaccination training, participants indicated a preference for online training (either interactive workshops or lectures) over in-person workshops or written materials. This could be due to an increased comfort in online meetings resulting from the COVID-19 pandemic, and or due to online courses being more flexible to attend than face-to-face trainings. This is important information to consider when planning training material and courses.

Second, among the professionals surveyed, physicians are the group that most frequently administers vaccination, engages in vaccination consultations, and encourages patients to follow the official vaccination recommendations. This can likely be explained by the sample being mainly composed of pediatricians (75%) and physicians in private practice (38, 39). Providing vaccination counseling is among their main roles in preventive medicine, and vaccination consultations are typically performed by pediatricians and general internists in Switzerland (14). Also, studies have shown that physicians see their role as that of an “advisor’’ or an “educator’’ who encourage patients to have their children vaccinated (40). Vaccine acceptance by HCPs is important as they are trusted sources of vaccination information for many. The early postgraduate period may an optimal time for HCPs to acquire the necessary knowledge to provide high quality vaccine recommendations (41, 42). For example, a 2020 US study examined HPV-related training, knowledge, and practices among residents in pediatrics, internal medicine, gynecology/obstetrics, and family medicine. The study found that pediatrics residents reported always recommending HPV vaccination significantly more than the other residents. This is important since pediatrics residents reported receiving evidence-based training on vaccine delivery, which was not the case for the other residents (43). In our study, nurses were the HCP group that most frequently reported answering questions from friends/acquaintances outside of work. Likewise, nurse respondents reported answering more questions from friends/acquaintances than from patients/clients at the workplace. This suggests that, in addition to “formal” vaccination counseling, which is most frequently done by physicians, “informal” vaccination counseling also exists and is important. We consider “informal” vaccination counseling to be when HCPs discuss vaccinations among their personal social networks, outside of work. Efforts to train HCPs about vaccination should not overlook the “informal” vaccination counseling aspect because HCPs serve as the face of the medical establishment in the eyes of many.

Potential explanations for lower numbers of recommendations coming from midwives may be due to them facing questions about vaccination less often and midwives tending to highlight the importance of patients/clients making the final decision by themselves (38–40). Other research has shown how physicians may internalize messages from public health authorities and colleagues which create expectations for “good doctors” to follow vaccine recommendations to achieve parent/patient adherence to official vaccination schedules. The same study provides evidence showing that when patients/clients do not agree with a physician’s vaccination recommendations, physicians may risk being perceived as “bad doctors” (14). Regarding HPV vaccine, many physicians, but few pharmacists and nurses, completely encouraged patients/clients to follow the official vaccination recommendations. This could be due to HCPs feeling uncomfortable addressing sexual topics (25) or HCPs being uncertain about the need for HPV vaccine (44, 45). Another potential explanation is that pharmacists and nurses may be less familiar with the vaccination schedule for adolescents compared to physicians. We included questions related to HPV vaccine because not much is known about HPV-vaccination recommendation practices of HCPs in Switzerland, and it is not clear which HCPs actually discuss HPV vaccination with target populations in practice (46, 47).

Third, less than half of the HCPs reported feeling comfortable counseling VH patients/clients, but HCPs may feel more comfortable as they gain professional experience. Between 15% (midwives) and 43% (physicians) HCPs reported being comfortable counseling VH patients/clients. Physicians were the group most comfortable in addressing VH in practice. However, if we consider that vaccination counseling forms a key preventive role of practicing physicians, and that they routinely counsel patients about vaccination, a 43% level of comfort is surprisingly low. This suggests an important need for additional training, both in terms of physician’s factual knowledge and communication approaches with VH patients/clients. In comparison, a US study from 2011 found that 84% of physicians felt comfortable addressing patient/client vaccination questions (16).

We identified several potential explanatory variables for HCPs’ comfort in addressing VH. These features were consistent across all HCP groups and included increasing professional experience, increased frequency by which HCPs encounter VH patients/clients, and having obtained additional training in CM. Each of these personal characteristics suggests diverse professional experience may assist HCPs with interacting with VH patients/clients, which previous research has shown to be a struggle for HCPs (13, 37). Most HCPs in this sample did not agree that it shows a lack of respect when patients/clients disagree with their vaccination recommendations. This is in contrast with a 2011 US study in which 35% of pediatricians reported that they felt it showed a lack of respect for their medical judgment when patients did not agree with their vaccination recommendations (16). None of the midwives agreed with this statement. Previous studies have also shown that midwives and nurses accept patients/clients decisions to vaccinate or not to vaccinate their child (40, 48). 2% or less of all HCP groups reported they preferred to no longer seeing VH patients/clients. This is in contrast to studies from the US (16–18) showing that the majority of physicians who responded to similar questions would prefer not to counsel VH patients and that they wished that VH patients would no longer come to their practice (49–51).

### Strengths and Limitations

A major strength of our study is the large sample size of a diverse group of HCP respondents across the country (N = 1,933). We provide novel insights into HCPs’ interest in additional training on vaccination knowledge and communication. This is an area that has received little previous research attention in Switzerland. The response rate is a limitation. The sample is not statistically representative, and response rates were lower in French- and Italian-speaking Switzerland. There was also likely selection bias, since HCPs interested in vaccination topics may have been more likely to participate (52). As with all survey methods, there was potential for participants to have recall bias in their response.

Although the questionnaire was piloted for content and clarity with a panel of experts representing each HCP group and four additional participants from each HCP group, the questionnaire was not validated with any statistical tests. Some questions left room for interpretation regarding the directionality of vaccination perspectives (i.e., favorable or unfavorable toward vaccination). For example, questions about disagreement (“Have any parents/patients stopped coming to you for consultations due to a disagreement with you about vaccination?”) do not specify if disagreements were around favorable or unfavorable vaccine attitudes. Future studies should address this by specifying the directionality of vaccination attitudes and by including rigorous validation of survey constructs.

Because the survey was administered during the COVID-19 pandemic, this likely influenced the interest and time availability of HCPs. The approval of coronavirus vaccinations during the survey period possibly influenced study results, but we are unable to determine what that influence may be with the data available.

### Conclusion

Our results indicate a high level of interest among HCPs in Switzerland for additional training on a variety of vaccination topics. In addition to knowledge-based vaccine education, training should include the development of communication skills. Knowledge and communication training for HCPs are essential for maintaining high vaccination coverage and for helping HCPs to feel more confident in counseling patients about vaccination.
