# Supplementary material for: Health Care Professionals’ Interest in Vaccination Training in Switzerland: A Quantitative Survey
Source: Int J Public Health. 2022 Nov 30;67:1604495. doi: 10.3389/ijph.2022.1604495 (PMC9749818; doi:10.3389/ijph.2022.1604495)
Supplement: Supplementary file 1 [file DataSheet1.PDF]

Supplementary

1.1 Survey of the physicians

Background

1) Gender
    a. Male
    b. Female
    c. Other
2) Age
    a. ≤ 30 years
    b. 31-40 years
    c. 41-50 years
    d. 51-60 years
    e. > 60 years
3) In which canton do you work?
4) Field of work
    a. General medicine (only adults)
    b. Family medicine (children and adults)
    c. Pediatrics
    d. Gynecology
    e. Infectious disease
    f. Other:
5) Place of work
    a. Hospital
    b. Practice
6) Position
    a. Resident
    b. Attending
7) What year did you pass the federal exam to practice medicine?
8) Do you have any additional accreditation or training in any discipline of complementary medicine?
    a. Yes ➔ Condition: Yes is selected. Skip to: Would you be interested in obtaining further training related to vaccination, and if yes, through what channels?
    b. No➔ Condition: No is selected. Skip to: What type of complementary medicine?

9) What type of complementary medicine?
   a. Anthroposophic medicine
   b. Traditional Chinese Medicine (TCM/Acupuncture)
   c. Homeopathy
   d. Phytotherapy
   e. Other:

Needs for training and continuing education

10) Would you be interested in obtaining further training related to vaccination, and if yes, through what channels?
    a. Yes, preferably through in-person workshops
    b. Yes, preferably online interactive workshops (i.e. via zoom or other platforms that allow for live seminars
    c. Yes, preferably through online lectures (live or not live)
    d. Yes, preferably through written material (journal articles, brochures)
    e. Other:
    f. No, not interested

11) How interested are you in obtaining additional knowledge/training on the following topic?

	**Not all interested**	**Slightly interested**	**Moderately interested**	**Very interested**
Vaccine safety				
Vaccine efficacy				
Vaccine preventable disease				
Vaccine ingredients				
Vaccine side effects				
Vaccine immunology				
Communication with vaccine hesitant parents/patients				
Shared decision-making about vaccination				
The Swiss Federal office of Public Health's vaccination schedule/ recommendations				
Other:				

Vaccination recommendations

12) To what extent do you encourage parents/patients to follow the official Swiss vaccination schedule for children? (1=not at all; 5=completely)
    a. 1 = not at all
    b. 2
    c. 3
    d. 4
    e. 5 = completely
    f. I am not familiar with the Swiss vaccination plan

13) To what extent do you encourage parents/patients to follow the official Swiss human papillomavirus (HPV) vaccination schedule for adolescents and young adults? (1=not at all; 5=completely)
    a. 1 = not at all
    b. 2
    c. 3
    d. 4
    e. 5 = completely
    f. I am not familiar with the Swiss vaccination plan
14) How satisfied are you with how the Swiss Federal Office of Public Health communicates the official Swiss vaccination schedule?
    a. Completely dissatisfied
    b. Somewhat dissatisfied
    c. Neither satisfied nor dissatisfied
    d. Somewhat satisfied
    e. Completely satisfied
    f. I am not familiar with the Swiss vaccination plan

Experience with vaccination administrations, vaccine hesitancy, delays and refusals

15) Do you administer vaccination?
    a. Yes ➔ Condition: Yes is selected. Skip to: How often do you vaccinate?
    b. No ➔ Condition: No is selected. Skip to: How often do you answer questions from parents/patients about vaccines?
16) How often do you vaccinate?
    a. At least once a day
    b. At least once a week
    c. At least once a month
    d. Less often than once a month
17) How often do you answer questions from parents/patients about vaccines?
    a. At least once a day
    b. At least once a week
    c. At least once a month
    d. Less often than once a month

18) How often do you answer questions about vaccinations from friends/acquaintances (i.e. outside of work)?
    a. At least once a day
    b. At least once a week
    c. At least once a month
    d. Less often than once a month
19) How often do you encounter vaccine hesitant* parents/patients? *defined as: somebody who has questions about the need, safety, and/or efficacy of certain or all vaccines
    a. At least once a day
    b. At least once a week
    c. At least once a month
    d. Less often than once a month

20) Have you ever refused care to parents/patients because he/she asked for vaccination later than recommended of refused vaccination altogether?
    a. Yes
    b. No
    c. I'm not sure
21) Have any parents/patients stopped coming to you for consultations due to a disagreement with you about vaccination?
    a. Yes
    b. No
    c. I'm not sure
22) Have any parents/patients decided to come to you for consultations due to a disagreement about vaccination with another healthcare provider?
    a. Yes
    b. No
    c. I'm not sure

Perspectives on vaccine hesitancy, delays and refusals

23) How comfortable are you counseling vaccine hesitant parents/patients?
    a. Comfortable
    b. Neither comfortable, nor uncomfortable
    c. Uncomfortable
    d. It does not matter, as I would prefer to no longer see these parents/patients
    e. Not applicable

24) When parents/patients disagree with my recommendations about vaccination, I feel it shows a lack of respect for my medical expertise.
    a. I agree
    b. Neither agree, nor disagree
    c. I disagree
    d. Not applicable

Individual, shared and mandated decisions

25) In my opinion, childhood vaccination, particularly the mumps, measles, and rubella (MMR), vaccine should be:
    a. Up to individuals
    b. A shared decision between parents/patients and doctors
    c. Mandated
    d. I do not wish to respond

26) In my opinion, decision concerning the annual influenza vaccination, should be:
    a. Up to individuals
    b. A shared decision between healthcare workers and employers
    c. Mandated
    d. I do not wish to respond

COVID-19

27) Has the corona pandemic changed parents'/patients' questions/concerns about vaccines, or willingness to vaccinate?
    a. Yes
    b. No
    c. Not sure
28) In what way?
    a. ____________

1.2 Survey of the pharmacists

Background

1) Gender
    a. Male
    b. Female
    c. Other
2) Age
    a. ≤ 30 years
    b. 31-40 years
    c. 41-50 years
    d. 51-60 years
    e. > 60 years
3) In which canton do you work?
4) Do you have any additional training or additional designations?
    a. Anamnesis in primary care
    b. Vaccination and blood collection
    c. Pharmaceutical support of institutions in the health care sector
    d. Consultation pharmacist outpatient medication prescription
    e. Integrated supply models
    f. Official Pharmacy
    g. Other:
5) Position
    a. Owner/manager of a pharmacy
    b. Employer
6) In which year did you complete your pharmaceutical studies (state examination)?
7) Do you have any additional accreditation or training in any discipline of complementary medicine?
    a. Yes ➔ Condition: Yes is selected. Skip to: How often do you vaccinate?
    b. No ➔Condition: No is selected. Skip to: What type of complementary medicine?
8) What type of complementary medicine?
    a. Anthroposophic medicine
    b. Homeopathy
    c. Phytotherapy
    d. Other:

Needs for training and continuing education

9) Would you be interested in obtaining further training related to vaccination, and if yes, through what channels?
   a. Yes, preferably through in-person workshops
   b. Yes, preferably online interactive workshops (i.e. via zoom or other platforms that allow for live seminars
   c. Yes, preferably through online lectures (live or not live)
   d. Yes, preferably through written material (journal articles, brochures)
   e. Other:
   f. No, not interested

10) How interested are you in obtaining additional knowledge/training on the following topic?

	**Not all interested**	**Slightly interested**	**Moderately interested**	**Very interested**
Vaccine safety				
Vaccine efficacy				
Vaccine preventable disease				
Vaccine ingredients				
Vaccine side effects				
Vaccine immunology				
Communication with vaccine hesitant parents/patients				
Shared decision-making about vaccination				
The Swiss Federal office of Public Health's vaccination schedule/ recommendations				
Other:				

Vaccination recommendations

11) To what extent do you encourage parents/patients to follow the official Swiss vaccination schedule for children? (1=not at all; 5=completely)
   a. 1 = not at all
   b. 2
   c. 3
   d. 4
   e. 5 = completely
   f. I am not familiar with the Swiss vaccination plan

12) To what extent do you encourage parents/patients to follow the official Swiss human papillomavirus (HPV) vaccination schedule for adolescents and young adults? (1=not at all; 5=completely)
    a. 1 = not at all
    b. 2
    c. 3
    d. 4
    e. 5 = completely
    f. I am not familiar with the Swiss vaccination plan
13) How satisfied are you with how the Swiss Federal Office of Public Health communicates the official Swiss vaccination schedule?
    a. Completely dissatisfied
    b. Somewhat dissatisfied
    c. Neither satisfied nor dissatisfied
    d. Somewhat satisfied
    e. Completely satisfied
    f. I am not familiar with the Swiss vaccination plan

Experience with vaccination administrations, vaccine hesitancy, delays and refusals

14) Do you administer vaccination?
    a. Yes ➔ Condition: Yes is selected. Skip to: How often do you vaccinate?
    b. No ➔ Condition: No is selected. Skip to: How often do you answer questions from parents/patients about vaccines?
15) How often do you vaccinate?
    a. At least once a day
    b. At least once a week
    c. At least once a month
    d. Less often than once a month
16) How often do you answer questions from parents/patients about vaccines?
    a. At least once a day
    b. At least once a week
    c. At least once a month
    d. Less often than once a month

17) How often do you answer questions about vaccinations from friends/acquaintances (i.e. outside of work)?
    a. At least once a day
    b. At least once a week
    c. At least once a month
    d. Less often than once a month

18) How often do you encounter vaccine hesitant* parents/patients? *defined as: somebody who has questions about the need, safety, and/or efficacy of certain or all vaccines
    a. At least once a day
    b. At least once a week
    c. At least once a month
    d. Less often than once a month

19) Have any parents/patients stopped coming to you for consultations due to a disagreement with you about vaccination?
    a. Yes
    b. No
    c. I'm not sure
20) Have any parents/patients decided to come to you for consultations due to a disagreement about vaccination with another healthcare provider?
    a. Yes
    b. No
    c. I'm not sure
21) How satisfied are you with what you are allowed to do in terms of vaccination?
    a. 1 = not at all
    b. 2
    c. 3
    d. 4
    e. 5 = completely
    f. I do not wish to respond

Perspectives on vaccine hesitancy, delays and refusals

22) How comfortable are you counseling vaccine hesitant parents/patients?
    a. Comfortable
    b. Neither comfortable, nor uncomfortable
    c. Uncomfortable
    d. It does not matter, as I would prefer to no longer see these parents/patients
    e. Not applicable
23) When parents/patients disagree with my recommendations about vaccination, I feel it shows a lack of respect for my medical expertise.
    a. I agree
    b. Neither agree, nor disagree
    c. I disagree
    d. Not applicable

Individual, shared and mandated decisions

24) In my opinion, childhood vaccination, particularly the mumps, measles, and rubella (MMR), vaccine should be:
    a. Up to individuals
    b. A shared decision between parents/patients and doctors
    c. Mandated
    d. I do not wish to respond

25) In my opinion, decision concerning the annual influenza vaccination, should be:
    a. Up to individuals
    b. A shared decision between health care workers and employers
    c. Mandated
    d. I do not wish to respond

COVID-19

26) Has the corona pandemic changed parents'/patients' questions/concerns about vaccines, or willingness to vaccinate?
    a. Yes
    b. No
    c. Not sure
27) In what way?
    a. ____________

1.3 Survey of the nurses

Background

1) Gender
    a. Male
    b. Female
    c. Other
2) Age
    a. ≤ 30 years
    b. 31-40 years
    c. 41-50 years
    d. 51-60 years
    e. > 60 years
3) In which canton do you work?
4) Field of work
    a. Adults (surgery, medicine, orthopedics)
    b. Pediatrics
    c. Adults and Pediatrics
    d. Psychiatry
    e. Other:
5) Place of work
    a. Hospital
    b. Rehabilitation Clinic
    c. Outpatient/Visiting nurse/Freelance
    d. Outpatient-employee (e.g. practice, MPA)
6) Position
    a. Work with patient contact
    b. Leadership/Management
    c. Education
    d. Research
7) Do you have any additional training or additional designations?
    a. Maternal counseling
    b. School nursing
    c. Pediatrics
    d. Operating Room
    e. Intensive Care
    f. Infection Prevention
    g. Master of Nursing Sciences

8) In which year did you complete your nursing training?

9) Do you have any additional accreditation or training in any discipline of complementary medicine?
    a. Yes ➔ Condition: Yes is selected. Skip to: Would you be interested in obtaining further training related to vaccination, and if yes, through what channels?
    b. No ➔ Condition: No is selected. Skip to: What type of complementary medicine

10) What type of complementary medicine?
    a. Anthroposophic medicine
    b. Traditional Chinese Medicine (TCM)
    c. Acupuncture
    d. Homeopathy
    e. Phytotherapy
    f. Other:

Needs for training and continuing education

11) Would you be interested in obtaining further training related to vaccination, and if yes, through what channels?
    a. Yes, preferably through in-person workshops
    b. Yes, preferably online interactive workshops (i.e. via zoom or other platforms that allow for live seminars
    c. Yes, preferably through online lectures (live or not live)
    d. Yes, preferably through written material (journal articles, brochures)
    e. Other:
    f. No, not interested

12) How interested are you in obtaining additional knowledge/training on the following topic?

	**Not all interested**	**Slightly interested**	**Moderately interested**	**Very interested**
Vaccine safety				
Vaccine efficacy				
Vaccine preventable disease				
Vaccine ingredients				
Vaccine side effects				
Vaccine immunology				
Communication with vaccine hesitant parents/patients				
Shared decision-making about vaccination				
The Swiss Federal office of Public Health's vaccination schedule/ recommendations				
Other:				

Vaccination recommendations

13) To what extent do you encourage parents/patients to follow the official Swiss vaccination schedule for children? (1=not at all; 5=completely)
    a. 1 = not at all
    b. 2
    c. 3
    d. 4
    e. 5 = completely
    f. I am not familiar with the Swiss vaccination plan
14) To what extent do you encourage parents/patients to follow the official Swiss human papillomavirus (HPV) vaccination schedule for adolescents and young adults? (1=not at all; 5=completely)
    a. 1 = not at all
    b. 2
    c. 3
    d. 4
    e. 5 = completely
    f. I am not familiar with the Swiss vaccination plan
15) How satisfied are you with how the Swiss Federal Office of Public Health communicates the official Swiss vaccination schedule?
    a. Completely dissatisfied
    b. Somewhat dissatisfied
    c. Neither satisfied nor dissatisfied
    d. Somewhat satisfied
    e. Completely satisfied
    f. I am not familiar with the Swiss vaccination plan

Experience with vaccination administrations, vaccine hesitancy, delays and refusals

16) Do you administer vaccination?
    a. Yes ➔ Condition: Yes is selected. Skip to: How often do you vaccinate?
    b. No ➔ Condition: No is selected. Skip to: How often do you answer questions from parents/patients about vaccines?
17) How often do you vaccinate?
    a. At least once a day
    b. At least once a week
    c. At least once a month
    d. Less often than once a month
18) How often do you answer questions from parents/patients about vaccines?
    a. At least once a day
    b. At least once a week
    c. At least once a month
    d. Less often than once a month
19) How often do you answer questions about vaccinations from friends/acquaintances (i.e. outside of work)?
    a. At least once a day
    b. At least once a week
    c. At least once a month
    d. Less often than once a month
20) How often do you encounter vaccine hesitant* parents/patients? *defined as: somebody who has questions about the need, safety and/or efficacy of certain or all vaccines
    a. At least once a day
    b. At least once a week
    c. At least once a month
    d. Less often than once a month
21) How satisfied are you with what you are allowed to do in terms of vaccination?
    a. 1 = not at all
    b. 2
    c. 3
    d. 4
    e. 5 = completely
    f. I do not wish to respond

Perspectives on vaccine hesitancy, delays and refusals

22) How comfortable are you counseling vaccine hesitant parents/patients?
    a. Comfortable
    b. Neither comfortable, nor uncomfortable
    c. Uncomfortable
    d. It does not matter, as I would prefer to no longer see these parents/patients
    e. Not applicable
23) When parents/patients disagree with my recommendations about vaccination, I feel it shows a lack of respect for my nursing competence.
    a. I agree
    b. Neither agree, nor disagree
    c. I disagree
    d. Not applicable

Individual, shared and mandated decisions

24) In my opinion, childhood vaccination, particularly the mumps, measles, and rubella (MMR), vaccine should be:
    a. Up to individuals
    b. A shared decision between parents/patients and doctors
    c. Mandated
    d. I do not wish to respond
25) In my opinion, decision concerning the annual influenza vaccination, should be:
    a. Up to individuals
    b. A shared decision between healthcare workers and employers
    c. Mandated
    d. I do not wish to respond

COVID-19

26) Has the corona pandemic changed parents'/patients' questions/concerns about vaccines, or willingness to vaccinate?
    a. Yes
    b. No
    c. Not sure
27) In what way?
    a. __________

1.4 Survey of the midwives

Background

1) Gender
    a. Male
    b. Female
    c. Other
2) Age
    a. ≤ 30 years
    b. 31-40 years
    c. 41-50 years
    d. 51-60 years
    e. > 60 years
3) In which canton do you work?
4) Place of work
    a. Labor ward
    b. Out-patient clinic
    c. Postnatal ward
    d. Prenatal ward
    e. Freelance
    f. Birthplace
    g. Practice
5) Position
    a. In training
    b. Employed
    c. Self-employed
    d. Management function
6) In which year did you complete your training as a midwife?
7) Do you have any additional accreditation or training in any discipline of complementary medicine?
    a. Yes ➔ Condition: Yes is selected. Skip to: Would you be interested in obtaining further training related to vaccination, and if yes, through what channels?
    b. No ➔Condition: No is selected. Skip to: What type of complementary medicine?

8) What type of complementary medicine?
    a. Anthroposophic medicine
    b. Traditional Chinese Medicine (TCM)
    c. Acupuncture
    d. Homeopathy
    e. Phytotherapy
    f. Other:

Needs for training and continuing education

9) Would you be interested in obtaining further training related to vaccination, and if yes, through what channels?
    a. Yes, preferably through in-person workshops
    b. Yes, preferably online interactive workshops (i.e. via zoom or other platforms that allow for live seminars
    c. Yes, preferably through online lectures (live or not live)
    d. Yes, preferably through written material (journal articles, brochures)
    e. Other:
    f. No, not interested

10) How interested are you in obtaining additional knowledge/training on the following topic?

	**Not all interested**	**Slightly interested**	**Moderately interested**	**Very interested**
Vaccine safety				
Vaccine efficacy				
Vaccine preventable disease				
Vaccine ingredients				
Vaccine side effects				
Vaccine immunology				
Communication with vaccine hesitant parents/patients				
Shared decision-making about vaccination				
The Swiss Federal office of Public Health's vaccination schedule/ recommendations				
Other:				

Vaccination recommendations

11) To what extent do you encourage parents/patients to follow the official Swiss vaccination schedule for children? (1=not at all; 5=completely)
    a. 1 = not at all
    b. 2
    c. 3
    d. 4
    e. 5 = completely
    f. I am not familiar with the Swiss vaccination plan
12) How satisfied are you with how the Swiss Federal Office of Public Health communicates the official Swiss vaccination schedule?
    a. Completely dissatisfied
    b. Somewhat dissatisfied
    c. Neither satisfied nor dissatisfied
    d. Somewhat satisfied
    e. Completely satisfied
    f. I am not familiar with the Swiss vaccination plan

Experience with vaccination administrations, vaccine hesitancy, delays and refusals

13) Do you administer vaccination?
    a. Yes ➔Condition: Yes is selected. Skip to: How often do you vaccinate?
    b. No ➔ Condition: No is selected. Skip to: How often do you answer questions from parents/patients about vaccines?
14) How often do you vaccinate?
    a. At least once a day
    b. At least once a week
    c. At least once a month
    d. Less often than once a month
15) How often do you answer questions from parents/patients about vaccines?
    a. At least once a day
    b. At least once a week
    c. At least once a month
    d. Less often than once a month

16) How often do you answer questions about vaccinations from friends/acquaintances (i.e. outside of work)?
    a. At least once a day
    b. At least once a week
    c. At least once a month
    d. Less often than once a month
17) How often do you encounter vaccine hesitant* parents/patients? *defined as: somebody who has questions about the need, safety, and/or efficacy of certain or all vaccines
    a. At least once a day
    b. At least once a week
    c. At least once a month
    d. Less often than once a month
18) How satisfied are you with what you are allowed to do in terms of vaccination?
    a. 1 = not at all
    b. 2
    c. 3
    d. 4
    e. 5 = completely
    f. I do not wish to respond

Perspectives on vaccine hesitancy, delays and refusals

19) How comfortable are you counseling vaccine hesitant parents/patients?
    a. Comfortable
    b. Neither comfortable, nor uncomfortable
    c. Uncomfortable
    d. It does not matter, as I would prefer to no longer see these parents/patients
    e. Not applicable
20) When parents/patients disagree with my recommendations about vaccination, I feel it shows a lack of respect for my professional competence.
    a. I agree
    b. Neither agree, nor disagree
    c. I disagree
    d. Not applicable

Individual, shared and mandated decisions

21) In my opinion, childhood vaccination, particularly the mumps, measles, and rubella (MMR), vaccine should be:
    a. Up to individuals
    b. A shared decision between parents/patients and doctors
    c. Mandated
    d. I do not wish to respond
22) In my opinion, decision concerning the annual influenza vaccination, should be:
    a. Up to individuals
    b. A shared decision between healthcare workers and employers
    c. Mandated
    d. I do not wish to respond
    e.

COVID-19

23) Has the corona pandemic changed parents'/patients' questions/concerns about vaccines, or willingness to vaccinate?
    a. Yes
    b. No
    c. Not sure
24) In what way?
    a. ____________
